# Two Cases of Succinate Dehydrogenase-Deficient Juvenile Gastric Gastrointestinal Stromal Tumor

**DOI:** 10.70352/scrj.cr.25-0143

**Published:** 2025-06-10

**Authors:** Daisuke Fujimoto, Taira Inutake, Junpei Takashima, Keizo Taniguchi, Hirotoshi Kobayashi

**Affiliations:** Department of Surgery, Teikyo University Hospital, Mizonokuchi, Kawasaki, Kanagawa, Japan

**Keywords:** SDH-deficient GIST, lymph node metastasis, juvenile

## Abstract

**INTRODUCTION:**

SDH-deficient GIST is a part of WT GIST that constitutes approximately 10% of gastric GISTs and has no mutation of proto-oncogene receptor tyrosine kinase or PDGFR-α. In this paper, we present 2 cases of juvenile WT gastric GIST with SDH deficiency: a woman who underwent initial surgical treatment in junior high school and subsequently underwent 2 surgical treatments, and a man with lymph node metastasis who underwent distal gastrectomy with lymphadenectomy.

**CASE PRESENTATION:**

The 1st case was a woman who was diagnosed with gastric GIST and underwent distal gastrectomy at another institution when she was in junior high school. And she was diagnosed with gastric GIST again at our institution after a close examination for anemia and underwent laparoscopic partial gastrectomy. Two years ago, a GIS revealed another multiple gastric GIST in the remnant stomach, and a total remnant gastrectomy with lymphadenectomy was performed. The 2nd case was a man who was diagnosed with gastric GIST after a thorough examination of the cause of anemia. A 30-mm gastric GIST was found in the antrum, and a distal gastrectomy with lymphadenectomy was performed in this case as well. Pathological findings showed a metastatic lymph node in the subpyloric region.

**CONCLUSIONS:**

Lymphadenectomy may be needed to improve the prognosis of juvenile GIST patients without distant metastasis because SDH-deficient GIST is more frequent in the younger generation, and SDH-deficient GIST has a higher frequency of lymph node metastasis.

## Abbreviations

α-SMA
: alpha-smooth muscle actin
DOG
: discovered on GIST
EUS
: endoscopic ultrasound
GIS
: upper gastrointestinal endoscopy
GIST
: gastrointestinal stromal tumor
HPF
: high-power fields
PDGFR-α
: platelet-derived growth factor receptor-alpha
SDH
: succinate dehydrogenase
SDHA
: succinate dehydrogenase subunit A
SDHB
: succinate dehydrogenase subunit B
SMT
: submucosal tumor
WT
: wild type

## INTRODUCTION

In GISTs, the proto-oncogene receptor tyrosine kinase (known as KIT, c-KIT, or CD117) is found in 75%–85% of the cases, and PDGFR-α mutations in about 10%. The remaining 10% have neither c-KIT nor PDGFR-α mutations, so-called WT-GISTs. Of these, 1%–2% are neurofibromatosis type 1 patient-related GISTs,^1)^ 2%–5% are GISTs with SDHB gene abnormalities,^2)^ 1% are GISTs with BRAF gene mutations,^3)^ and a few percent have no mutations in any of the above genes. Many GISTs occur in older adults, but SDH-deficient GISTs usually present before the age of 40 years in females and have characteristic features including multinodular, multifocal, and occasionally lymph node metastasis.^4)^ Standard GIST therapies (tyrosine kinase inhibitors) are less efficacious in SDH-deficient GISTs.^5)^

Herein, we report 1 male and 1 female SDH-deficient GIST who underwent robotic gastrectomy with systematic lymphadenectomy similar to gastrectomy for gastric cancer after preoperative suspicion of WT-GIST.

## CASE PRESENTATION

**Fig. 1:**
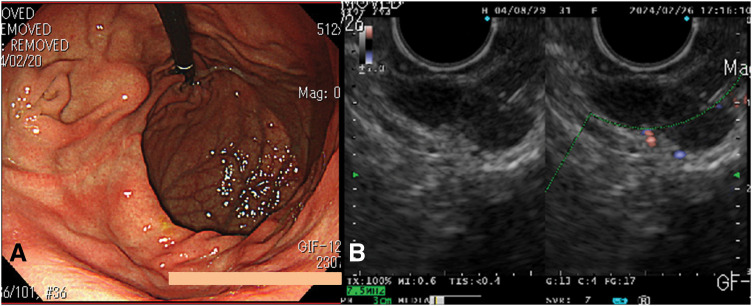
Representative images from abdominal examinations. (**A**) The upper gastrointestinal endoscopy revealed a submucosal tumor on the lesser curvature wall of the remnant stomach. (**B**) The endoscopic ultrasound showed 3 hypoechoic masses with a maximum diameter of 16 mm, which were suspected to be submucosal tumors.

**Fig. 2:**
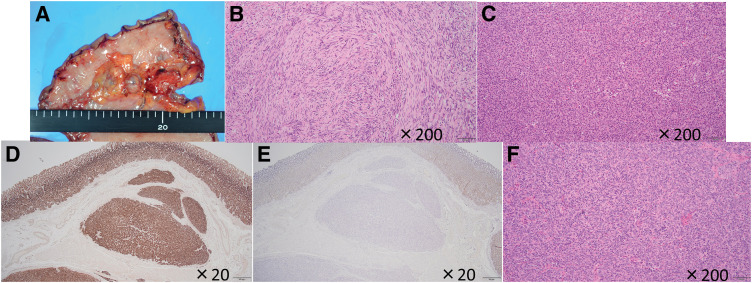
Pathological findings. (**A**) Gross findings of the resected remnant stomach. Four masses were seen on the lesser curvature side of the remnant stomach. (**B** and **C**) Microscopic findings showed spindle-shaped and epithelioid cells (original magnification ×200). (**D**) Positive staining with succinate dehydrogenase subunit A immunostaining (original magnification ×20). (**E**) Loss of staining with succinate dehydrogenase subunit B immunostaining (original magnification ×20). (**F**) The pathological findings of the 2nd surgery (original magnification ×20).

The 1st case is a 31-year-old woman who underwent laparoscopic distal gastrectomy and Billroth I reconstruction for a gastric GIST at the age of 15 at another institution (the pathology findings at that time were diagnosed at a different institution and therefore cannot be presented); at age 28, she underwent partial gastrectomy for a heterochronic GIST at our institution; at yearly follow-up, GIS revealed a new SMT in the remnant stomach (**Fig. 1A**). EUS also showed a well-multinodular growth tumor on the remnant stomach, related to the muscularis propria (**Fig. 1B**). The SMT was pathologically diagnosed as a GIST based on findings from ultrasound-guided fine-needle aspiration biopsy: epithelioid growth pattern cells, immunopathological DOG1-positive,α-SMA-negative, and S-100-negative during routine diagnostic workup. No clinical findings suggesting dissemination and distant metastasis were detected using contrast-enhanced CT and ^18^F-fluorodeoxyglucose positron emission tomography. Her family history seemed to be irrelevant, since no other cases of GISTs, paragangliomas, or pheochromocytomas were reported. The patient’s parents and younger sister are in good health, and no related neoplastic lesions were found. Hence, suspecting a WT-GIST from a juvenile woman’s GIST with multiple and heterochronic lesions, we performed a robotic total remnant gastrectomy with regional lymph node dissection, including the supra-pancreatic region. No intraoperative or postoperative complications occurred. With an unremarkable postoperative recovery, the postoperative hospital stay was 8 days. The gross examination of the specimen showed that there were 4 tumors with a maximum diameter of 13-mm, well-demarcated intramural masses located in the remnant stomach (**Fig. 2A**). In terms of pathological examination, the tumor was composed of spindle cells grouped in short fascicles and whorls as well as sheets of epithelioid cells (**Fig. 2B** and **2C**). One mitosis per 50 HPF was found. No metastasis was found in the lymph nodes that were dissected. Based on these pathological findings, the tumor was diagnosed as a very low risk according to the modified Fletcher classification. Modified Immunohistochemistry examination showed a strong expression of CD117 and DOG1. SDHB stains showed loss of expression, but SDHA stains showed positive (**Fig. 2D** and **2E**). The pathology of the GIST resected in the 2nd surgery at age 28 was also shown. Although no search was carried out for SDH defects at that time, the pathological finding was composed of round or polygonal cells with abundant clear or acidophilic cytoplasm and indistinct cell borders (**Fig. 2F**). One year and 4 months after surgery, she is surviving and recurrence-free.

**Fig. 3:**
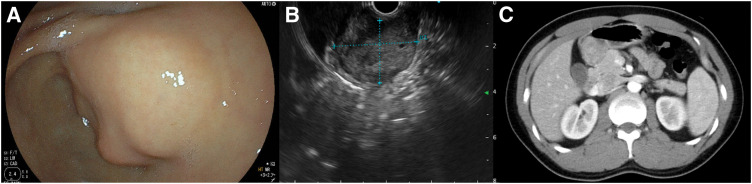
Representative images from abdominal examinations. (**A**) The upper gastrointestinal endoscopy showed a submucosal tumor on the posterior wall of the antrum. (**B**) The endoscopic ultrasound showed a hypoechoic mass in the 4th layer with clear borders but multifocal and somewhat heterogeneous interior. (**C**) Computed tomography showed a mass with contrast effect in the antrum. No clearly swelling lymph nodes were seen.

**Fig. 4:**
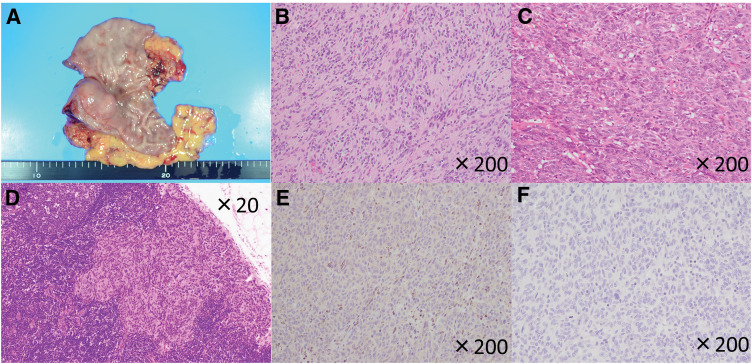
Pathological findings. (**A**) Gross findings of the resected sample. Two masses were seen on the posterior wall of the antrum. (**B** and **C**) Microscopic findings showed spindle-shaped and epithelioid cells (original magnification ×200). (**D**) Microscopic findings showed epithelioid cells in lymph node (original magnification ×200). (**E** and **F**) Loss of staining with succinate dehydrogenase subunit A and succinate dehydrogenase subunit B immunostaining (original magnification ×200).

The 2nd case was a 32-year-old male. The GIS was performed for a close examination of anemia and revealed a lobulated SMT with a dip located in the antrum (**Fig. 3A**). An ulcer was found at the apex of the SMT, which was diagnosed as the cause of his anemia. The EUS also showed a well-multiloculate tumor on the antrum, related to the muscularis propria (**Fig. 3B**). The SMT was pathologically diagnosed as a GIST based on findings from ultrasound-guided fine-needle aspiration biopsy: dense epithelioid growth pattern cells without myxomatous stroma, immunopathological CD117-positive, DOG1-positive, α-SMA-negative, and S-100-negative during routine diagnostic workup. No clinical findings suggesting dissemination and distant metastasis were detected using contrast-enhanced CT (**Fig. 3C**). His family history also seemed to be irrelevant, since no other cases of GISTs, paragangliomas, or pheochromocytomas were reported. Although this patient was male, we suspected a WT-GIST because of his young age, and we performed a robotic distal gastrectomy with D1+ lymphadenectomy.^6)^ No intraoperative or postoperative complications occurred. With an unremarkable postoperative recovery, the postoperative hospital stay was 8 days. The gross examination of the specimen showed that there were 4 tumors with a maximum diameter of 30-mm, well-demarcated intramural masses located in the antrum (**Fig. 4A**). More than 10 mitoses per 50 HPF were found. Based on these pathological findings, the tumor was diagnosed as high risk according to the modified Fletcher classification. In terms of pathological examination, the tumor was composed of spindle cells grouped in short fascicles and whorls as well as sheets of epithelioid cells, too (**Fig. 4B** and **4C**). Among the lymph nodes that were dissected, 2 metastases were found in the subpyloric lymph node (**Fig. 4D**).^7)^ Immunohistochemistry examination showed a strong expression of CD117 and DOG1. SDHA and SDHB stains showed loss of expression (**Fig. 4E** and **4F**). One year after surgery, he is surviving and recurrence-free.

## DISCUSSION

GISTs are the most common mesenchymal neoplasm of the gastrointestinal tract, with an annual incidence of approximately 10–15 cases per million, and have a predilection for patients in their 50s–60s, with no gender difference.^8)^ The most commonly located site of GIST is the stomach, followed by the small intestine and colon.^9)^ Approximately 85% of GISTs are mutually exclusive in the CD117 or PDGFR-α gene.^10,11)^ Complete surgical resection is a mainstay of treatment for GIST of the stomach.^12,13)^ SDH-deficient GISTs represent the largest population of WT-GISTs, and the frequency of those was reported to be approximately 7.4%–7.6%.^14–16)^ SDH-deficient GIST is more common in women under 40 years of age and exhibits clinical and pathological characteristics, such as primary localization in the stomach, multinodular growth, an epithelioid phenotype, and a common lymph vascular invasion.^17)^

Primary tumor location, mitotic rate, and tumor size correlate with the prognosis of patients with GIST.^18)^ The maximum is the number of 10 mitoses per 50 HPF, and the lower limit is 1 mitosis per 50 HPF. Tumors classified as high or low grade are those that fall above and below this cut-off threshold.^19)^ Another significant predictive feature is tumor size; patients with tumors ≤2, 2.1–5.0, 5.1–10, and >10 cm are classified as low, intermediate, and high-risk categories, respectively.^19)^ Additionally, lymph node metastasis was suggested to independently predict decreased overall survival for adult patients with GIST.^20)^ In GIST, liver metastasis and peritoneal dissemination are the most common forms of metastasis, and regional lymph node metastasis is extremely infrequent; therefore, lymph node dissection is not routinely performed.^21)^ Even when lymph nodes are suspected to be metastasis, systemic lymph node dissection has no clinical significance, and pick-up dissection of the relevant lymph node is considered sufficient.^22)^ But some patient populations, including SDH-deficient GIST in children, adolescents, and young adults, have been shown to have an increased risk of lymph node metastasis,^23)^ and there is no literature reporting on the sentinel lymph node studies or the detection of metastatic lymph nodes by CT in GIST. Therefore, we believe it is difficult to pick up only the metastatic lymph node, and the only way to remove the metastatic lymph node in SDH-deficient GIST is by dissecting the regional lymph node to ensure complete curative resection.

In our 2 cases, both patients did not show significant lymph node swelling on preoperative CT. The 1st case underwent robotic-remnant total gastrectomy with suprapancreatic lymphadenectomy, and the 2nd case underwent robotic-distal gastrectomy with D1+ lymphadenectomy. The 1st case showed no lymph node metastasis, but the 2nd case showed 2 metastases in the No. 6 lymph node of the subpyloric region. Histopathological examination revealed GIST with intricate proliferation of short spindle-shaped and round tumor cells in continuity with lymph node structures, and the possibility of peritoneal dissemination was ruled out. Many cases of lymph node metastasis have been reported in cases with liver metastasis or peritoneal metastasis or in high-risk cases in the Fletcher classification.^13)^ If lymph node metastasis is present without either liver metastasis or peritoneal metastasis, lymph node dissection may improve the patient’s prognosis.^24)^ Even in the 2nd case in which lymph node metastasis was detected, the maximum lymph node diameter of the No. 6 lymph node on preoperative CT was less than 5 mm, which was not considered to be significant swelling, and the diagnosis of clinical N0 was made. However, there are cases of lymph node metastasis despite clinical N0, and the addition of lymphadenectomy may improve the prognosis,^24)^ so it is important to decide based on sufficient informed consent considering functional preservation and curative resection. Therefore, even if SDH-deficient GIST is diagnosed with preoperative biopsy, it is difficult to determine whether a total gastrectomy with lymphadenectomy should be performed. Since SDH-deficient GIST is characterized by a large number of young patients and slow growth,^25)^ we believe that it is better to perform gastrectomy with lymphadenectomy with functional preservation under the condition that postoperative follow-up is very careful, except in cases where radical resection is clearly difficult without a total gastrectomy. And, in the National Comprehensive Cancer Network guideline of GIST, resection of enlarged lymph nodes should be considered in patients with SDH-deficient GIST, but there is no mention of prophylactic lymph node dissection or extent of dissection.^25)^ Furthermore, the European Society for Medical Oncology clinical practice guidelines for GIST state that mutational analysis inclusions in the diagnostic work-up of all GISTs should be considered standard practice (with the possible exclusion of <2 cm non-rectal GISTs) and immunohistochemistry assessment of SDH deficiency prior to treatment initiation may be useful in making treatment decisions.^26)^ The NCCN guidelines for GIST state that if a preoperative diagnosis of SDH-deficient GIST is made, germline testing for SDH mutations would be indicated. Patients with SDH germline mutations are at risk of paraganglioma, that is, at risk for intraoperative hypertension and postoperative hypotension and hypoglycemia; 24-hour urine testing is recommended prior to surgery.^25)^ In both cases presented here, preoperative thoracoabdominal CT showed no paraganglioma, and both intraoperative and postoperative courses were good, but after the postoperative diagnosis of SDH-deficient GIST, a search for germline testing SDH mutations revealed that 1 patient had SDHB mutations in the germline. We believe that when a WT-GIST is suspected in a juvenile patient, it is necessary to include a search for SDH deficiency.

## CONCLUSIONS

In conclusion, we believe that lymphadenectomy may be needed to improve the prognosis of juvenile GIST patients without distant metastasis because SDH-deficient GIST are more frequent in the younger generation, and SDH-deficient GIST have a higher frequency of lymph node metastasis than CD117/PDGFR-α-mutant GIST. However, further study of the usefulness of systematic lymphadenectomy is needed.

## DECLARATIONS

### Funding

This work was supported in part by the Japan Society for the Promotion Science (KAKENHI 22K07260 to DF).

### Authors’ contributions

DF wrote the initial draft of the manuscript.

DF, KT, and TI performed the surgery, and DF and JT followed up with these patients.

HK critically reviewed the manuscript.

All authors have read and approved the final manuscript.

All authors agree to be responsible for aspects of the study.

### Availability of data and materials

The datasets of this case report are available from the corresponding author upon reasonable request.

### Ethics approval and consent to participate

This work does not require ethical considerations or approval.

### Consent for publication

Informed consent was obtained from the patients for the publication of this case report and accompanying images.

### Competing interests

The authors declare that they have no competing interests.
